# Medical College Note

**Published:** 1898-07

**Authors:** 


### MEDICAL COLLEGE NOTE.

THE medical department of Niagara University has been consolidated with the medical department of the University of Buffalo. The final details of the union were arranged June 21, 1898, although it appears that the proposition has been under consideration for some time past by leading members of both faculties. The additions to the teaching corps of Buffalo resultant are as follows: Thomas Lothrop, M. A., M. D., Ph. D., honorary professor of obstetrics; Alvin A. Hubbell, M. D., Ph. D., clinical professor of ophthalmology and otology; Henry D. Ingraham, M. D., clinical professor of gynecology and pediatrics; Herman Mynter, M. D., clinical professor of surgery; Herbert Mickle, M. D., adjunct professor of clinical surgery; Carlton C. Frederick, M. Sc., M. D., clinical professor of gynecology; John A. Miller, M. Sc., M. A., Ph. D., associate professor of organic chemistry and toxicology, Eugene A. Smith, M. D., adjunct professor of clinical surgery; Henry C. Buswell, M. D., adjunct professor of principles and practice of medicine; W. Scott Renner, M. D., C. M., clinical professor of laryngology and rhinology; Walter D. Greene, M. D., clinical professor of genito-urinary diseases; Harry A. Wood, M. D., clinical professor of insanity; Earl P. Lothrop, A. B., M. D., adjunct clinical professor of obstetrics; Alfred E. Diehl, A. M., M. D., adjunct clinical professor of dermatology; George Roberts, M. D., adjunct professor of pathology; Emil S. Tobie, A. B., M. D., instructor in materia medica; Wm. K. O'Callaghan, M. D., clinical assistant in obstetrics.

The change is made in the interests of harmony and in accordance with the spirit of the age which insists that there are too many medical colleges. It is hoped and believed that it will prove beneficial to all individuals concerned and to the profession in general.

